# Microvascular Disease Associates with Larger Osteocyte Lacunae in Cortical Bone in Type 2 Diabetes Mellitus

**DOI:** 10.1002/jbm4.10832

**Published:** 2023-10-27

**Authors:** Sebastian Zanner, Elliott Goff, Samuel Ghatan, Eva Maria Wölfel, Charlotte Ejersted, Gisela Kuhn, Ralph Müller, Morten Frost

**Affiliations:** ^1^ Molecular Endocrinology Department, Department M Odense University Hospital Odense Denmark; ^2^ Clinical Institute University of Southern Denmark Odense Denmark; ^3^ Institute for Biomechanics ETH Zurich Zurich Switzerland; ^4^ Department of Internal Medicine Erasmus MC University—Medical Center Rotterdam Rotterdam The Netherlands; ^5^ Steno Diabetes Centre Odense OUH Odense Denmark

**Keywords:** ANALYSIS/QUANTITATION OF BONE, BIOMECHANICS, BONE QCT/MICRO‐CT, CELLS OF BONE, OSTEOCYTES, ORTHOPEDICS

## Abstract

Clinical studies indicate that microvascular disease (MVD) affects bone microstructure and decreases bone strength in type 2 diabetes mellitus (T2D). Osteocytes are housed in small voids within the bone matrix and lacunae and act as sensors of mechanical forces in bone. These cells regulate osteoclastic bone resorption and osteoblastic bone formation as well as osteocytic perilacunar remodeling. We hypothesized that MVD changes morphometric osteocyte lacunar parameters in individuals with T2D. We collected iliac crest bone biopsies from 35 individuals (10 female, 25 male) with T2D with MVD (15%) or without MVD (21%) with a median age of 67 years (interquartile range [IQR] 62–72 years). The participants were included based on c‐peptide levels >700 pmol L^−1^, absence of anti‐GAD65 antibodies, and glycated hemoglobin (HbA1c) levels between 40 and 82 mmol mol^−1^ or 5.8% and 9.7%, respectively. We assessed osteocyte lacunar morphometric parameters in trabecular and cortical bone regions using micro‐computed tomography (micro‐CT) at a nominal resolution of 1.2 μm voxel size. The cortical osteocyte lacunar volume (Lc.V) was 7.7% larger (*p* = 0.05) and more spherical (Lc.Sr, *p* < 0.01) in the T2D + MVD group. Using linear regression, we found that lacunar density (Lc.N/BV) in trabecular but not cortical bone was associated with HbA1c (*p* < 0.05, *R* ^2^ = 0.067) independently of MVD. Furthermore, Lc.V was larger and Lc.Sr higher in the center than in the periphery of the trabecular and cortical bone regions (*p* < 0.05). In conclusion, these data imply that MVD may impair skeletal integrity, possibly contributing to increased skeletal fragility in T2D complicated by MVD. © 2023 The Authors. *JBMR Plus* published by Wiley Periodicals LLC on behalf of American Society for Bone and Mineral Research.

## Graphical abstract

In this article, we are using micro‐CT imaging to demonstrate a larger cortical osteocyte lacunar volume in patients with type 2 diabetes mellitus (T2D) and microvascular disease (MVD) compared with patients without MVD. Furthermore, we observe an association of glucose control and trabecular lacunar density independently of MVD. Finally, our data indicate a general low cortical lacunar volume in T2D when compared with previous published articles with healthy participants.

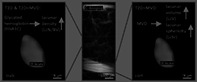

## Introduction

Type 2 diabetes mellitus (T2D) affects more than 450 million individuals and is predicted to affect an increasing number of people in the years ahead.^(^ ^1^ ^)^ Insulin insensitivity and inadequate insulin secretion in T2D cause hyperglycemia, which may lead to microvascular disease (MVD), including neuropathy, nephropathy, and retinopathy.^(^ ^2^, ^3^ ^)^ In addition, fragility fractures are more commonly reported in T2D compared with individuals without diabetes, despite normal or elevated bone mass measured by dual energy X‐ray absorptiometry (DXA).^(^ ^4^, ^5^, ^6^, ^7^ ^)^ Fracture risk is not homogeneously distributed across individuals with T2D. In a recent Swedish registry study, fracture risk was higher in patients who were on insulin treatment, with low physical activity, a body mass index (BMI) below 25 kg m^−2^, and long‐standing disease duration but lower in most (55%) individuals with T2D than in age‐ and sex‐matched background population.^(^ ^8^ ^)^ Other factors are also considered to increase fracture risk in T2D, including increased risk of falls, poor glycemic control, and diabetes complications, including MVD.^(^ ^9^ ^)^

Clinical studies based on high‐resolution peripheral quantitative computed tomography (HR‐pQCT) scans have shown an increased cortical porosity in T2D,^(^ ^10^ ^)^ which appears to be most pronounced in T2D patients with MVD.^(^ ^11^ ^)^ Reduced skeletal blood flow resulting from peripheral vascular disease is associated with higher cortical porosity in individuals with T2D,^(^ ^12^ ^)^ supporting that vascular dysfunction may influence bone structure. The mechanisms underlining higher cortical porosity in T2D complicated by MVD remain unknown and may include several factors, such as impaired recruitment of osteogenic cell types, tissue hypoxia, or restricted availability of nutrients in the cortical bone.

Histological investigations have demonstrated lower bone remodeling in T2D.^(^ ^4^, ^13^ ^)^ In the process of bone remodeling, osteoclasts resorb bone, which is followed by osteoblastic bone formation. A fraction of the osteoblasts become embedded in the bone matrix, where they terminally differentiate to osteocytes. The mature osteocytes develop processes that span the entire bone matrix and connect osteocytes, cells on the bone surface, and the lumen of the vasculature^(^ ^14^ ^)^ to form the lacunocanalicular network. Through this network, mechanical strains are directed by fluid pressure to the osteocytes, which orchestrate bone remodeling. After mechanical unloading, osteocytes secrete sclerostin, which inhibits bone formation by blocking Wnt signaling.^(^ ^15^ ^)^ Although serum sclerostin levels and insulin sensitivity were not associated in healthy and prediabetic men,^(^ ^16^ ^)^ sclerostin levels are increased in overt T2D.^(^ ^17^ ^)^ This indicates that hyperglycemia changes the function of osteocytes and the lacunocanalicular network, which may lead to lower bone remodeling and higher cortical porosity in T2D. Importantly, glucose increases *SOST* expression and sclerostin content in cultured osteocyte‐like IDG‐SW3 cells, which was validated in vivo and ex vivo in streptozotocin‐induced hyperglycemic rats.^(^ ^18^ ^)^ Further, it was demonstrated that sclerostin directly promotes the resorption of the perilacunar matrix, a process called osteocytic osteolysis.^(^ ^19^, ^20^ ^)^ These findings support that T2D may influence osteocyte function and subsequently bone remodeling.

Besides bone remodeling through osteoclasts and osteoblasts, osteocytes can also change the lacunar morphology through perilacunar remodeling, eg, to adapt to mechanical forces,^(^ ^21^ ^)^ which may be changed in pathological conditions such as T2D and MVD. In mice, lactation induces osteocytic osteolysis, which increases the sensitivity of osteocytes due to a larger osteocyte lacunar volume (Lc.V).^(^ ^22^ ^)^ With age, Lc.V decreases and lacunar sphericity (Lc.Sr) increases,^(^ ^23^ ^)^ which may lead to a reduced ability to sense mechanical loading. The consequences of the development of MVD in T2D to osteocyte lacunae morphology have not been explored. We speculate that impaired blood flow may lead to hypoxia and subsequently a metabolic shift of the osteocyte toward anerobic glycolysis, which could result in acidification of the lacunae and enhanced osteocytic osteolysis, resulting in larger lacunae. We further separately evaluated peripheral lacunae close to bone surface (<20 μm) and central lacunae (>20 μm) to account for possible differences in age of the lacunae, nutrient availability, mechanical loading, and proximity to the vasculature.

Previous studies have demonstrated increased cortical porosity in T2D; however, these data are based on HR‐pQCT scans, which cannot capture osteocyte lacunar size and shape.^(^ ^24^, ^25^, ^26^ ^)^ We hypothesize that MVD as observed in T2D changes iliac crest morphometric osteocyte lacunar parameters in T2D patients. Therefore, the aim of this study was to investigate cortical and trabecular osteocyte lacunar morphology in T2D individuals with and without MVD using high‐resolution micro‐CT.

## Materials and Methods

### Study design and population

This is a cross‐sectional study in adult individuals with T2D with and without MVD. Individuals were prospectively included in the study between May 2017 and March 2020 at the endocrine outpatient clinics at Odense University Hospital (OUH) and Kolding hospital, Denmark. All procedures were conducted in accordance with the Declaration of Helsinki and were approved by local ethics committee (ID: 21/21773). Written informed consent was obtained from all participants. Men and postmenopausal women with a history of T2D >5 years were included. The inclusion criteria also covered plasma c‐peptide levels >700 pMol and the absence of antibodies against glutamic acid decarboxylase (anti‐GAD65) at any time point after the T2D diagnosis. Candidates for this study were excluded in case of monogenic or secondary diabetes, if glycated hemoglobin (HbA1c) was above 75 mmol mol^−1^ at the time of enrollment, or if insulin treatment was instigated within the first 2 years after the diagnosis. In addition, candidates with known metabolic bone or calcium disorders, chronic kidney disease (creatinine >90 μMol), or liver dysfunction (aspartate transaminase >3× upper limit) were excluded. Candidates were excluded if they were on any medical treatment known to influence bone metabolism, eg, systemic corticosteroids. Information on fracture history including trauma mechanism (low‐ or high‐energy trauma) were registered at time of inclusion. Furthermore, at enrollment it was assessed whether the participants had MVD, which was defined by the presence of a clinical diagnosis of either nephropathy, neuropathy, retinopathy, or a combination of the aforementioned. According to the MVD status, participants were grouped into the T2D group or the type 2 diabetes complicated by MVD group (T2D + MVD).

### DXA scans and bone biopsy

Areal bone mineral density (BMD) at the lumbar spine (L_1_ to L_4_, lumbar spine BMD), hip (total hip BMD), and nondominant hip region (femoral neck BMD) were measured using DXA scans (Hologic Discovery, Waltham, MA, USA). The coefficient of variation (CV) is 1.5% at both the spine and the hip at our bone unit (OUH, Odense, Denmark). Female reference ranges were used to calculate *T*‐scores in both men and women to test osteoporosis status. In addition, trans‐iliac crest bone biopsies were collected using a modified Bordier trephine (inner diameter: 8 mm). The biopsies were subsequently dehydrated in ethanol and embedded in poly methyl methacrylate (PMMA) at −20°C.

### Sample preparation and micro‐CT analysis

**Fig. 1:**
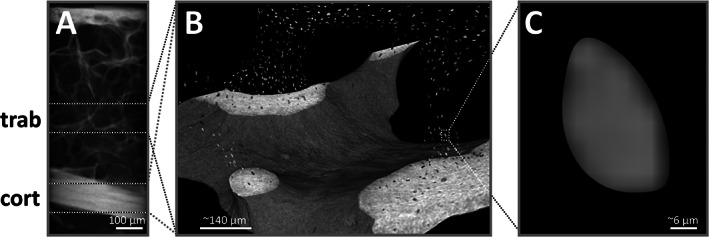
(*A*) Overview scans of 35 iliac crest bone biopsies from two diabetes mellitus (T2D) patients with (*n* = 20) or without microvascular disease (MVD; *n* = 15) were scanned at 6 μm resolution. (*B*) Two stacks (1.09 mm) were selected for high‐resolution scans (1.2 μm nominal resolution). (*C*) The image data was segmented, and tissue parameters and single osteocyte lacunar parameters were determined.

We scanned the specimens using micro‐computed tomography (micro‐CT 50, Scanco Medical AG, Brüttisellen, Switzerland). A previously validated cutting and image segmentation process was used to identify osteocyte lacuna characteristics.^(^ ^27^ ^)^ In brief, disks with the bone biopsies of a diameter of 25 mm and a thickness of 3.8 mm were cut with a diamond blade (SCAN‐DIA Minicut 40, SCAN‐DIA GmbH & Co. Kg, Hagen, Germany) to a rectangular block and turned with a conventional lathe (Schaublin 102, Bevilard, Switzerland) to obtain a cylinder with a diameter of 3.8 mm ± 0.05 mm. This cylinder was placed into a custom‐made sample holder and scanned at 6 μm nominal resolution, followed by two high‐resolution (HR‐micro‐CT) scans with 1.2 μm nominal resolution covering a cortical and a trabecular bone region. The 3D image stacks were acquired using 72 μA current, 4 W power, 55 kVp energy, 1.5‐second integration time, level 6 data averaging, and 1500 X‐ray projections (Fig. 1). Osteocyte lacunae were segmented using image inversion after applying an individualized threshold based on the distribution of tissue mineral density values of each sample. Morphometric parameters of segmented lacunae were calculated with a custom Python script^(^ ^27^ ^)^ (3.7.1, Python Software Foundation, Wilmington, DE, USA) used in combination with the software XamFlow (Lucid Concepts AG, Zurich, Switzerland) as previously described in Goff and colleagues.^(^ ^27^ ^)^ By this, the following lacunar morphometric parameters were assessed: bone volume/tissue volume (BV/TV), cortical thickness (Ct.Th), osteocyte lacunar number (Lc.N) and density (Lc.N/BV), osteocyte Lc.V, osteocyte lacunar oblateness (Lc.O),^(^ ^28^ ^)^ and osteocyte lacunar sphericity (Lc.Sr).^(^ ^29^ ^)^ For the lacunar parameters, local lacunar parameters were used, meaning that apart from Lc.N, the data were presented as means of each bone biopsy, which results in an equal power per sample but varying impact of each lacuna due to different Lc.N per sample. Additionally, Lc.V and Lc.Sr were assessed individually for peripheral (<20 μm from bone surface) and the central (>20 μm from bone surface) regions of the of the bone compartments.

### Statistical analyses

We applied linear regression models to test whether osteocyte lacunae characteristics were different between the T2D and T2D + MVD groups and to determine their relationship with clinical characteristics such as MVD, HbA1c, and T2D disease duration. Regression models were adjusted for age and sex of the individuals. Comparisons between peripheral and central osteocyte lacunar parameters of the same sample were tested using Wilcoxon tests. Data distribution was assessed using histograms, and non‐normally distributed data was log10 transformed. All analyses were performed using R (Version 3.5.1), and results were considered significant for *p* ≤ 0.05.

## Results

### Clinical characteristics including prevalence of osteoporosis

Thirty‐five individuals were included (15 female and 20 male) of which 43% (*n* = 15, 5 female and 10 male) had MVD. The median age of the participants was 67.0 years (interquartile range [IQR] 63.8–72.0), and the mean HbA1c was 56 mmol mol^−1^ (SD: 9.48) or 7.3% (SD: 0.87). All participants were diagnosed with T2D for at least 5 years, with a mean of 16.09 years (SD: 7.67). Overall, clinical differences between participants with T2D with and without MVD were not observed. BMD at the lumbar spine, femoral neck, and total hip were similar in both groups (Table 1). Six participants were diagnosed with osteoporosis based on either a *T*‐score of −2.5 or lower (2 in the T2D group and 2 in the T2D + MVD group) or an osteoporotic vertebral or hip fracture (2 in the T2D + MVD group).

**Table 1:** Clinical Characteristics of the Study Participants

	Total (*n* = 35)	T2D (*n* = 20)	T2D + MVD (*n* = 15)	*p* Value
Age (years)	67 (IQR 62–72)	68 (IQR 65–72)	63 (IQR 62–73)	0.66
Sex				
Male	71%	75%	67%	
Female	29%	25%	33%	0.59
BMI (kg m^−2^)	30 (IQR 26–34)	29 (IQR 26–31)	33 (IQR 29–34)	0.26
Weight (kg)	92 (IQR 79–103)	89 (IQR 79–103)	92 (IQR 80–107)	0.53
Height (cm)	173 (IQR 165–180)	174 (IQR 165–180)	171 (IQR 165–180)	0.82
Diabetes duration (years)	16 (SD 7.7)	15 (SD 6.8)	18 (SD 8.7)	0.30
HbA1c (mmol mol^−1^)	56.2 (SD 9.5)	54.5 (SD 8.6)	58.5 (SD 10.0)	0.22

Clinical bone parameters	Total (*n* = 34)	T2D (*n* = 20)	T2D + MVD (*n* = 14)	
Femoral neck BMD (g cm^−2^)	0.808 (SD 0.15)	0.807 (SD 0.14)	0.808 (SD 0.16)	0.94
Total hip BMD (g cm^−2^)	1.005 (SD 0.17)	0.995 (SD 0.16)	1.019 (SD 0.17)	1.00
Lumbar spine BMD (g cm^−2^)	1.065 (SD 0.16)	1.059 (SD 0.17)	1.073 (SD 0.14)	0.94

*Note*: Data presented as median (IQR) or mean (SD).

Abbreviations: BMI = body mass index; BMD = bone mineral density; HbA1c = glycated hemoglobin; IQR = interquartile range; MVD = microvascular disease; T2D = type 2 diabetes mellitus.

### Bone biopsy parameters

**Fig. 2:**
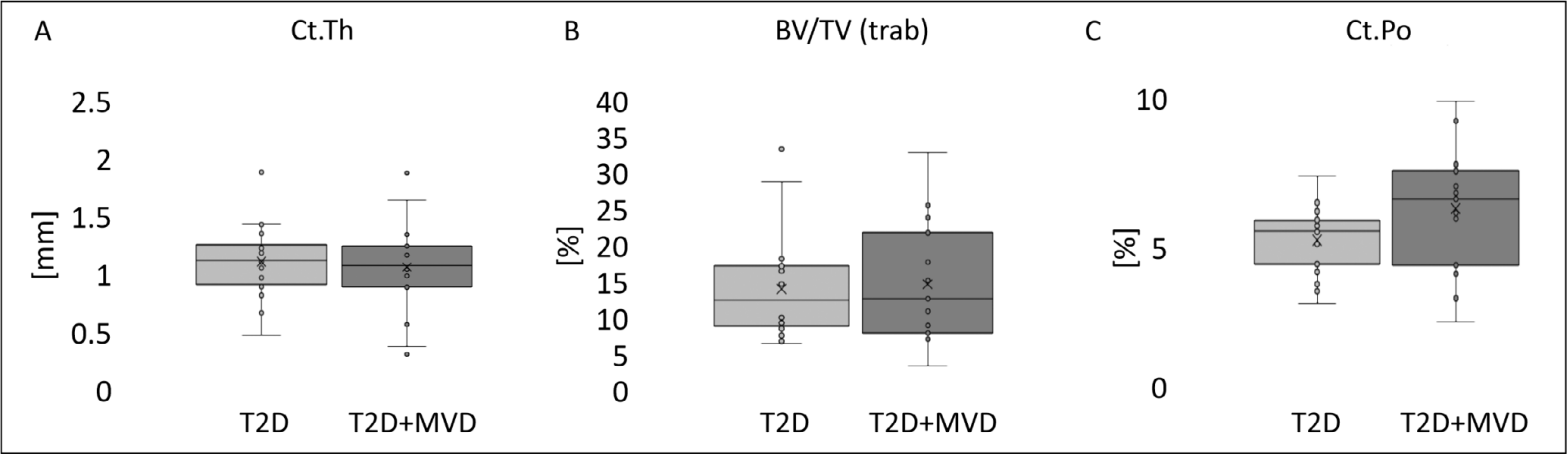
Micro‐CT scans of iliac crest bone from two diabetes mellitus (T2D) patients with or without microvascular disease (MVD). (*A*) Trabecular bone tissue volume (bone volume/tissue volume [BV/TV(trab)]): T2D: 15.1 ± 6.8%, T2D + MVD: 15.7 ± 7.9%, *p* = 0.82, *n* = 35. (*B*) Cortical thickness (Ct.Th): T2D: 1.16 ± 0.24 mm, T2D + MVD: 1.15 ± 0.24 mm, *p* = 0.91, *n* = 33. (*C*) Cortical porosity (Ct.Po): T2D: 5.27 ± 1.22, T2D + MVD: 5.92 ± 2.01%, *p* = 0.31, *n* = 33. No differences between groups (determined by 2‐tailed, heteroscedastic *t* tests).

**Fig. 3:**
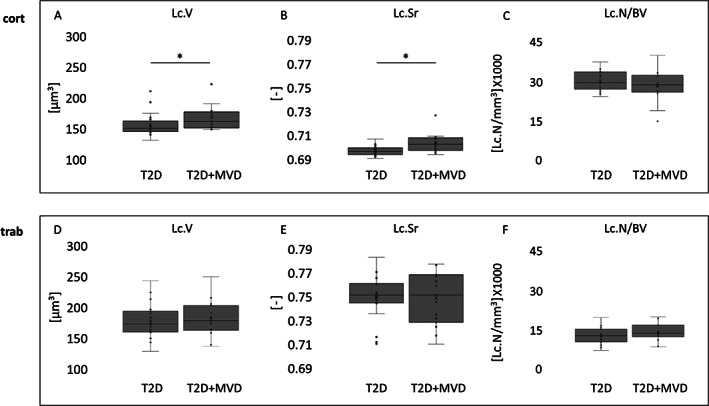
Iliac crest osteocyte lacunar parameters. (*A*) Cortical (cort) lacunar volume (Lc.V), *p* = 0.054, *n* = 33. (*B*) Lacunar sphericity (Lc.Sr): *p* < 0.005, *n* = 33. (*C*) Cort Lc.N/BV, *p* = 0.24, *n* = 33. (*D*) Trabecular (trab) Lc.V, *p* = 0.60, *n* = 35, (*E*) Trab Lc.Sr, *p* = 0.97, *n* = 35. (*F*) Trab Lc.N/BV, *p* = 0.23, *n* = 34. *p ≤ 0.05 (determined by linear regression analysis controlled for age and sex).

Because of the lack of cortical regions in two biopsies, 33 biopsies (20 T2D, 13 T2D + MVD) were used for cortical and 35 biopsies (20 T2D, 15 T2D + MVD) for trabecular analyses. Micro‐CT scans showed that cortical and trabecular BV/TV, Ct.Th, and cortical porosity (Ct.Po) were not significantly different in the T2D and T2D + MVD groups (Fig. 2). Osteocyte lacunae were evaluated using HR‐micro‐CT scans (summarized in Table 2) and revealed a 7.7% larger Lc.V in cortices of the T2D + MVD group compared with the T2D group. This accounts on average for an increase in volume of 12 μm^3^ per lacuna (169 μm^3^ in T2D + MVD versus 160 μm^3^ in T2D, *p* = 0.05). We did not observe group differences in Lc.V in the trabecular bone (173 μm^3^ versus 178 μm^3^, *p* = 0.61, *n* = 35). Furthermore, cortical Lc.Sr was significantly higher in the T2D + MVD group compared with the T2D group (0.703 versus 0.697, respectively, *p* = 0.005). There were no group differences between T2D + MVD and T2D in trabecular Lc.N/BV (13,704/mm^3^ versus 12,778/mm^3^, respectively) and Lc.O (−0.356 versus −0.330, respectively) or cortical Lc.N/BV (28,507/mm^3^ versus 29,413/mm^3^, respectively) and Lc.O (−0.365 versus −0.370, respectively) (Fig. 3). We further analyzed if the lacunar parameters Lc.V, Lc.Sr, and Lc.N/BV differ with the sex of the participants independently of MVD. We identified trends of larger cortical Lc.V in men (165 mm^3^ versus 151 mm^3^, *p* = 0.09) and a lower trabecular Lc.N/BV in men (12,659/mm^3^ versus 14,797/mm^3^, *p* = 0.08) (Supplemental Fig. S1 and Supplemental Table S1). Differences in one sex group according to MVD status were not compared because of the low number of individuals in the female group.

**Table 2:** Iliac Crest Cortical and Trabecular Morphometric Osteocyte Lacunar Parameters

Trab	T2D (*n* = 20)	T2D + MVD (*n* = 15)	Beta	SE	*p* Value
Lc.N (#)	440,578	360,863	‐	‐	‐
Lc.V (μm^3^)	173 (IQR 160–193)	178 (IQR 163–203)	0.0277	0.0534	0.61 (log)
Lc.Sr (−)	0.752 (IQR 0.745–0.761)	0.752 (IQR 0.729–0.768)	−0.0003	0.0073	0.97 (log)
Lc.N/BV (#/mm^3^)	12,778 (IQR 10,488–15,211)	13,704 (IQR 12,412–16,770)	0.0983	0.0808	0.23 (log)
Lc.O (−)	−0.330 (IQR −0.349– −0.313)	−0.356 (IQR −0.376– −0.318)	−0.0121	0.0091	0.19

Cort	T2D (*n* = 20)	T2D + MVD (*n* = 13)			
Lc.N (#)	4,739,141	2,389,627	‐	‐	‐
Lc.V (μm^3^)	151 (IQR 146–163)	162 (IQR 152–178)	0.0780	0.0390	**0.05** (log)
Lc.Sr (−)	0.697 (IQR 0.694–0.700)	0.703 (IQR 0.708–0.708)	0.0094	0.0031	**0.005** (log)
Lc.N/BV (#/mm^3^)	29,413 (IQR 26,889–33,317)	28,507 (IQR 25,762–32,084)	−0.0810	0.0670	0.24 (log)
Lc.O (−)	−0.369 (IQR −0.374– −0.362)	−0.365 (IQR −0.371– −0.358)	−0.0811	0.0673	0.24 (log)

Trab center	T2D (*n* = 20)	T2D + MVD (*n* = 15)			
Lc.N (#)	282,024	227,282	‐	‐	‐
Lc.V (μm^3^)	192 (IQR 169–222)	203 (IQR 165–229)	0.0176	0.0656	0.79 (log)
Lc.Sr (−)	0.769 (IQR 0.758–0.778)	0.7692 (IQR 0.738–0.792)	−0.00070	0.0090	0.94 (log)
Lc.O (−)	−0.306 (IQR −0.336– −0.282)	−0.346 (IQR −0.371– −0.304)	−0.0202	0.0165	0.23 (log)

Trab peripheral	T2D (*n* = 20)	T2D + MVD (*n* = 15)			
Lc.N (#)	158,554	133,581	‐	‐	‐
Lc.V (μm^3^)	142 (IQR 135–153)	139 (IQR 130–162)	−0.0285	0.0349	0.42 (log)
Lc.Sr (−)	0.728 (IQR 0.724–0.740)	0.728 (IQR 0.707–0.744)	−0.000066	0.00062	0.92 (log)
Lc.O (−)	−0.356 (IQR −0.377– −0.347)	−0.372 (IQR −0.394– −0.348)	−0.0059	0.0094	0.54 (log)

Cort center	T2D (*n* = 20)	T2D + MVD (*n* = 13)			
Lc.N (#)	4,191,349	2,064,612	‐	‐	‐
Lc.V (μm^3^)	154 (IQR 148–167)	166 (IQR 157–185)	0.0825	0.0407	**0.05** (log)
Lc.Sr (−)	0.697 (IQR 0.694–0.701)	0.704 (IQR 0.698–0.709)	0.0103	0.0034	**0.005** (log)
Lc.O (−)	−0.370 (IQR −0.375– −0.362)	0.366 (IQR −0.372– −0.357)	0.0064	0.0053	0.23 (log)

Cort peripheral	T2D (*n* = 20)	T2D + MVD (*n* = 13)			
Lc.N (#)	522,254	325,015	‐	‐	‐
Lc.V (μm^3^)	124 (IQR 121–129)	135 (IQR 123–140)	0.0528	0.0290	0.08 (log)
Lc.Sr (−)	0.695 (IQR 0.693–0.699)	0.697 (IQR 0.694–0.703)	0.0044	0.0024	0.08 (log)
Lc.O (−)	−0.365 (IQR −0.368– −0.360)	−0.361 (IQR −0.365– −0.357)	0.0032	0.0032	0.33 (log)

*Note*: Data presented in medians. The *p* values were determined using linear regression models controlled for age and sex.

Abbreviations: BMD = bone mineral density; HbA1c = glycated hemoglobin; IQR = interquartile range; Lc.N = lacunar number; Lc.N/BV = lacunar density; Lc.O = lacunar oblateness; Lc.Sr = lacunar sphericity; Lc.V = lacunar volume; MVD = microvascular disease; T2D = type 2 diabetes mellitus.

### Association between lacunar characteristics with HbA1c and disease duration

**Fig. 4:**
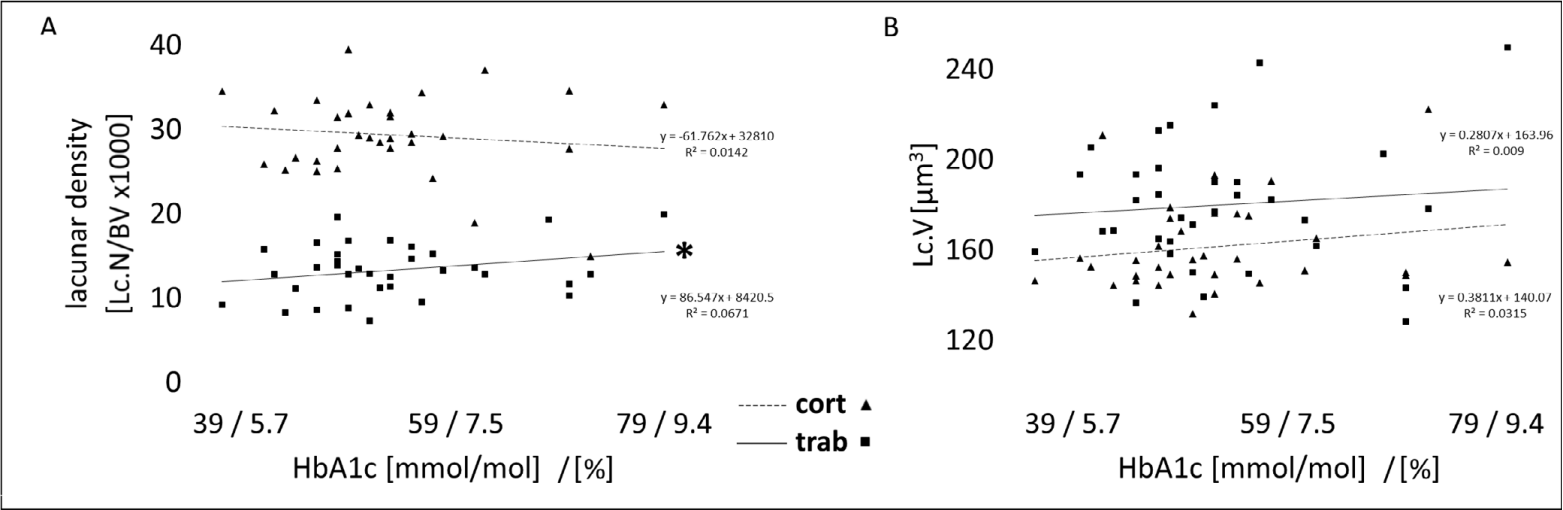
Glycated hemoglobin (HbA1C) correlated with (*A*) lacunar density (Lc.N/BV) or (*B*) lacunar volume (Lc.V) in cortical (cort, triangles) and trabecular (trab, squares) bone regions of iliac crest bone from T2D patients. *p < 0.05, *n* = 34.

Next, we assessed the association of HbA1c and disease duration to lacunar characteristics independent of MVD status. HbA1c was associated with trabecular but not cortical bone Lc.N/BV (*p* < 0.05, *n* = 34). The linear regression showed an increase of 87 Lc.N/BV for every mmol mol^−1^ (0.09%) increase of HbA1c (Fig. 4*A*). HbA1c was not associated with Lc.V in trabecular or cortical bone (Fig. 4*B*).

### Lc.V and lacunar sphericity in subregions of cortical and trabecular bone

**Fig. 5:**
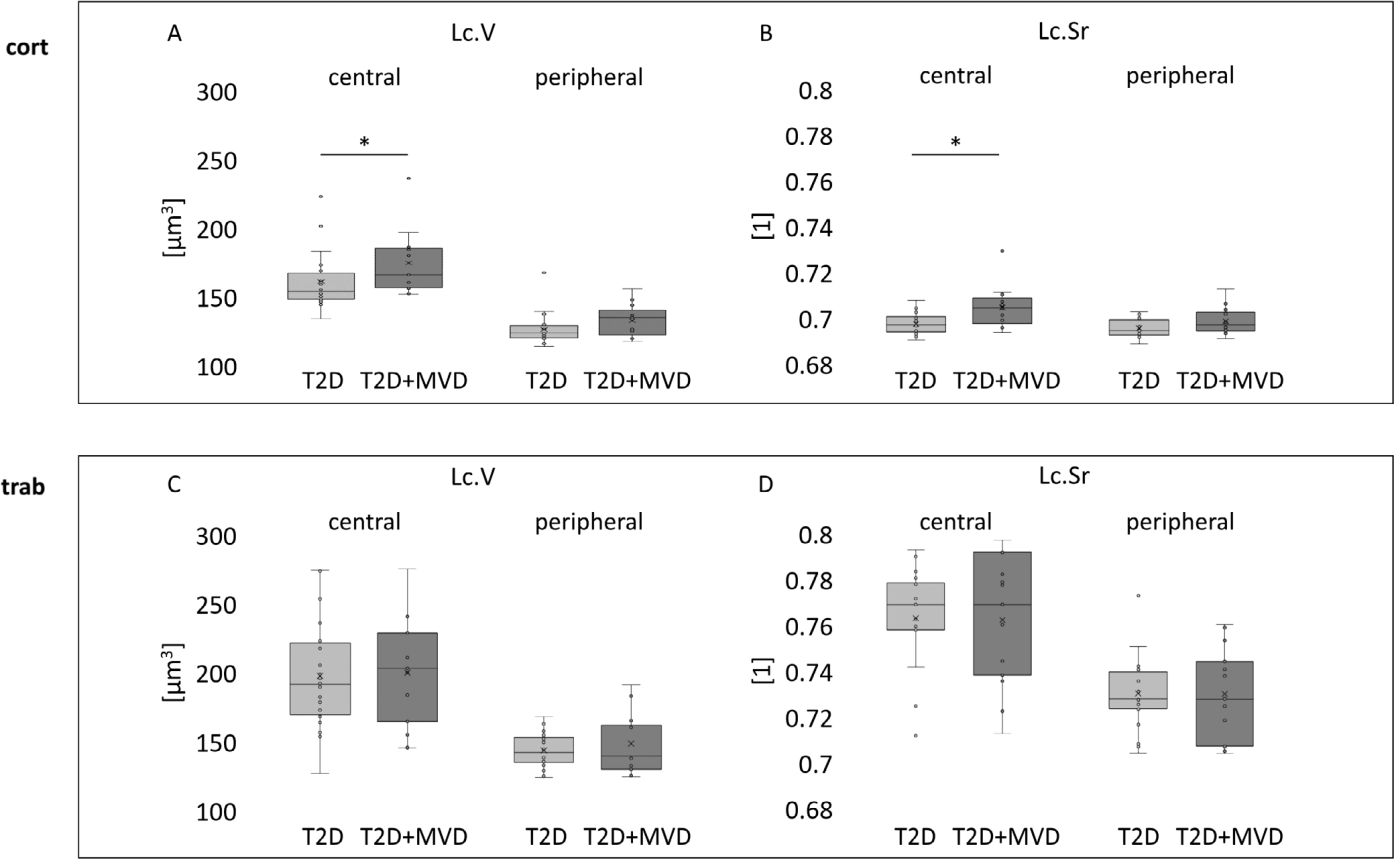
(*A*) Lacunar parameters in central (>20 μm from bone surface) and peripheral bone regions (<20 μm from bone surface). Data are presented in cortical (cort, *B*, *C*) and trabecular (trab, *D*, *E*) bone. Lacunar volume (Lc.V) and lacunar sphericity (Lc.Sr) in central bone are significantly higher in the T2D + MVD group compared with the T2D group (*p* = 0.052 and *p =* 0046, respectively).

Finally, we compared lacunar parameters from osteocyte lacunae close to the surface (peripheral, <20 μm) and the remaining lacunae in trabecular and cortical bone (central). Central cortical Lc.V and Lc.Sr were significantly larger in the T2D + MVD group versus the T2D group (166 μm^3^ versus 154 μm^3^ and Lc.Sr 0.704 versus 0.697. *p* = 0.05 and *p* < 0.01, respectively) but not in the peripheral region (135 μm^3^ versus 124 μm^3^, respectively, and Lc.Sr 0.697 versus 0.695, respectively, *p* = 0.08 and *p* = 0.08, respectively) (Fig. 5).

Similar differences were not observed in trabecular bone (Table 2). Also, our data showed that lacunae in central regions of cortical and trabecular bone presented with larger Lc.V and higher Lc.Sr than lacunae in peripheral regions (cort central Lc.V 166 μm^3^ versus cort peripheral Lc.V 129 μm^3^ and central Lc.Sr 0.700 versus peripheral Lc.Sr 0.697, *p* < 0.005 and *p* < 0.05, respectively; trab central Lc.V 199 μm^3^ versus trabecular peripheral Lc.V 146 μm^3^, trab central Lc.Sr 0.763 versus trab peripheral Lc.Sr 0.730, *p* < 0.001 and *p* < 0.001, respectively) (Table 3).

**Table 3:** Comparison of Central and Peripheral Lacunae

	Peripheral	Center	*p* Value
Cort Lc.N (#)	872,807	6,255,961	‐
Trab Lc.N (#)	292,135	509,306	‐
Cort Lc.V (μm^3^)	129 (IQR 121–136)	166 (IQR 152–180)	2.13e − 13 (log)
Trab Lc.V (μm^3^)	146 (IQR 133–160)	199 (IQR 168–223)	8.38e − 11 (log)
Cort Lc.Sr (−)	0.697 (IQR 0.693–0.700)	0.700 (IQR 0.695–0.704)	0.037 (log)
Trab Lc.Sr (−)	0.730 (IQR 0.719–0.741)	0.763 (IQR 0.719–0.781)	2.24e − 07 (log)

*Note*: Data presented in median and interquartile range. The *p* values were determined by Wilcoxon test.

Abbreviations: IQR = interquartile range; Lc.N = lacunar number; Lc.Sr = lacunar sphericity; Lc.V = lacunar volume.

## Discussion

Using high‐resolution micro‐CT imaging, we show that osteocyte lacunar characteristics including cortical Lc.V and cortical Lc.Sr were increased in individuals with T2D complicated by MVD. Furthermore, we observed a positive correlation between Hb1Ac and Lc.N/BV in trabecular but not cortical bone independently of the MVD status. Finally, we demonstrated regional differences in Lc.V and Lc.Sr between central and peripheral lacunae both in trabecular and cortical bone.

Two studies using the same technique to assess iliac crest bone tissue from healthy premenopausal women reported similar lacunae volumes in trabecular bone with median Lc.V of 178 μm^3^ (IQR 159–197) and 197 μm^3^ (IQR 185–221), respectively.^(^ ^27^, ^29^ ^)^ In both studies, lacunae volumes in the cortical bone specimens were larger with median Lc.Vs of 223 μm^3^ (IQR 189–245)^(^ ^27^ ^)^ and 282 μm^3^ (IQR 228–303),^(^ ^29^ ^)^ respectively. In postmenopausal women, Akhter and colleagues reported trabecular Lc.Vs of 188 μm^3^ (IQR 172–215) and cortical Lc.Vs of 245 μm^3^ (IQR 211–275). Although we observed similar median trabecular Lc.V of 172 μm^3^ (IQR 160–193) for the T2D group and 178 (IQR 163–203) for the T2D + MVD group, cortical Lc.V were smaller in this study with 151 μm^3^ (IQR 146–263) for the T2D group and 163 μm^3^ (IQR 152–178) for the T2D + MVD group. Although direct comparisons are challenging, these data indicate that cortical Lc.V may be smaller in individuals with T2D. Importantly, this study included elderly men and postmenopausal women, suggesting that dissimilarities in Lc.V measured in the present study and those conducted in pre‐ and postmenopausal women could also be explained by age and sex in addition to the presence of T2D. Although there were no statistically significant sex‐specific differences in any of the outcomes, cortical Lc.V and Lc.Sr tended to be higher in men. The MVD status was not considered in these analyses because of a low number of samples in each group.

Although lacunae volumes have been reported in other studies, comparisons are particularly challenging because of differences in imaging techniques, resolution, sample acquisition and segmentation, and sample sites. For example, one large study based on iliac crest bone using synchrotron micro‐CT reported mean cortical Lc.Vs of 538 μm^3^ (SD ± 31) in women and 523 μm^3^ (SD ± 39), whereas trabecular Lc.V was not reported. Bach‐Gansmo and colleagues did not find an association of morphometric lacunar parameters with age but a decrease of lacunar density with age.^(^ ^30^ ^)^ Furthermore, diseases and their treatments may also influence the lacunae morphology. Increased Lc.V have been observed in rodents on parathyroid hormone (PTH) treatment,^(^ ^31^ ^)^ in patients with vitamin D deficiency,^(^ ^32^ ^)^ and during lactation,^(^ ^33^ ^)^ suggesting that different mechanisms may influence Lc.V.

The osteocyte lacunar densities identified in this investigation are similar to some^(^ ^34^, ^35^, ^36^ ^)^ but not all^(^ ^29^, ^30^, ^37^ ^)^ previous reports. Akhter and colleagues^(^ ^29^ ^)^ reported higher Lc.N/BV in trabecular bone compared with cortical bone in iliac crest biopsies, whereas we observed an almost 2× higher Lc.N/BV in cortical than in trabecular bone in our study population. Although Lc.N/BV appeared not to be affected by MVD in T2D, we did observe an association between HbA1c and trabecular but not cortical Lc.N/BV, which might be due to a higher bone turnover and a higher connection of the bone surface to the bone marrow of trabecular bone than cortical bone. The higher bone turnover may drive a faster change in Lc.N/BV potentially as a compensation of hyperglycemia‐induced loss of mechanosensitivity.

Independent of MVD, we also observed higher Lc.V and Lc.Sr in central than in peripheral bone regions of the cortical and trabecular region. A key role of osteocytes is the inhibition of bone formation during mechanical unloading, which is achieved by the expression of sclerostin, a strong inhibitor of the Wnt signaling pathway that promotes bone formation.^(^ ^15^ ^)^ In normal conditions, mechanical loading results in a drop of sclerostin expression to favor osteoblastogenesis and thereby bone formation. In a mouse experiment, it was demonstrated that an increase in Lc.V is associated with a higher suppression of *SOST* in bone after mechanical loading.^(^ ^22^ ^)^ This indicates that osteocytes may utilize osteocytic osteolysis to increase the mechanosensitivity. It is possible that osteocyte lacunae in the central part of the bone experience different mechanical loading than those in the peripheral regions of the bone, which may lead to changes in osteocyte Lc.V to compensate for different mechanical stimuli. Larger Lc.V in the T2D + MVD group than in the T2D group without the complication was found in the central but not in the peripheral part of the cortical bone region, indicating that group differences in the entire cortical region were driven by the central lacunae.

The vasculature is closely connected to the lacunocanalicular network with canaliculi connecting both microenvironments, the vascular system and the lacunocanalicular network.^(^ ^14^ ^)^ Therefore, changes in vasculature may be transferred to the osteocytes and may contribute to perilacunar remodeling. Although the mechanisms leading to larger Lc.V in T2D complicated by MVD remain to be determined, it may be that increased blood glucose causes the previously reported elevated sclerostin levels in T2D,^(^ ^17^, ^38^, ^39^ ^)^ which could induce osteocytic osteolysis, resulting in larger Lc.V. In murine and human cultured osteocyte‐like cells, it was demonstrated that sclerostin directly upregulates key enzymes for resorption.^(^ ^20^ ^)^ This finding was further validated in vivo in mouse bone by enlarged Lc.V after elevating sclerostin levels.^(^ ^19^ ^)^ Further, metabolic changes due to hypoxia and acidification may drive Lc.V enlargement in MVD. Long‐standing hyperglycemia is a risk factor for MVD in T2D, and larger Lc.V may also serve as an adaption to a cellular loss of mechanosensing abilities caused by elevated glucose levels in the T2D + MVD group.

A recent meta‐analysis identified and increased cortical thickness in the distal radius and tibia, as well as increased cortical porosity in the radius but not in the tibia in adult T2D patients assessed using HR‐pQCT at a resolution of 82 μm.^(10)^ Using the same technique, Shanbhogue and colleagues^(^ ^11^ ^)^ showed that individuals with T2D complicated by MVD had a higher cortical porosity at the radius and tended to have a higher cortical porosity at the tibia than healthy controls and T2D patients without MVD. Although these studies indicate that mechanical loading may prevent expansion of cortical porosity at the tibia, the latter finding suggests that MVD affects cortical porosity even at weight‐bearing sites.

This is supported by higher cortical porosity in the distal tibia in T2D patients with peripheral vascular disease.^(^ ^12^ ^)^ Using micro‐CT scans with a resolution of 6 μm, we did not observe an increase in cortical porosity in iliac crest biopsies in T2D patients with MVD. Importantly, this does not preclude presence of higher cortical porosity in MVD based on pores with a size above approx. 160 μm. We speculate that MVD impairs angiogenesis, resulting in a lower number of newly formed blood vessels and basal membrane thickening that expands blood vessels. Although purely speculative, the absence of higher cortical porosity in the T2D + MVD group in the present study could be explained by higher density of small blood vessels in the T2D group and larger blood vessels in the T2D + MVD group, resulting in a similar degree of cortical porosity using micro‐CT scans but larger cortical porosity in a MVD group if assessed using HR‐pQCT scans.

Further analyses of the osteocyte lacunar morphometry revealed an increased Lc.Sr in the T2D + MVD group. Because Lc.Sr is defined by the lacunar surface area (Lc.SA) and the Lc.V, Lc.Sr may influence Lc.V or vice versa. Lc.Sr is considered to be an indicator for the mechanosensing ability of osteocytes, especially in weight‐bearing bone. Thus, the osteocyte may reshape the osteocyte lacuna to decrease or increase the impact of mechanical loading on the osteocyte. In weight‐bearing bone, this may lead to an elongation of the lacunae along the applied load direction to decrease fluid pressure.^(^ ^40^ ^)^ In our study, cortical Lc.Sr was higher in the T2D + MVD group, potentially indicating a loss of mechanosensitivity of the osteocyte and a counter‐regulatory perilacunar remodeling to increase the impact of mechanical stimuli. Importantly, our investigation was based on bone tissue from a non‐weight‐bearing site. It remains to be investigated if MVD in T2D affects osteocyte sphericity in bone that is subjected to larger mechanical loading than in the iliac crest.

This study has several limitations. The present study included a cohort of 35 iliac crest biopsies from well‐characterized adult men and women with T2D. However, because of the absence of healthy matched controls, we were limited to comparing our results to published data sets. Furthermore, the cross‐sectional study design precluded assessment of changes in factors that may influence osteocyte lacunar morphology such as glucose control. As stated above, inverse relationships between vitamin D levels and lacunae volumes have been reported. Vitamin D levels were not available in the present investigation; therefore, we cannot exclude the possibility that vitamin D deficiency may have influenced the results. Although the imaging technique used allowed for an in‐depth assessment of the osteocyte lacunae, we were unable to test the impact of T2D and MVD on the osteocyte canaliculi or perilacunar matrix composition, which should be the focus of future investigations. Because of the lack of actual assessment of bone vasculature, we were unable to determine if MVD is present in the bone microenvironment or represents a marker for long‐standing uncontrolled blood glucose. Finally, we should mention that MVD is a composite of three clinical findings, and more investigations are needed to determine if the impact of MVD on bone is driven by neuropathy, nephropathy, changes in the vasculature, or a combination.

In conclusion, our investigation demonstrated a larger Lc.V and a higher Lc.Sr in adults with T2D + MVD than in individuals with T2D without overt MVD. Furthermore, we observed an association of HbA1c with higher Lc.N/BV in trabecular bone, which together indicate that diabetes may change morphometric lacunar parameters and lacunar density. Finally, we report smaller cortical Lc.V in our study population, which is also reported with aging.^(^ ^41^ ^)^ Jointly, these investigations suggest that diabetic MVD may affect osteocyte function, which could contribute to diabetic bone fragility.

## Author Contributions

**Sebastian Zanner:** Data curation; formal analysis; investigation; methodology; project administration; visualization; writing – original draft; writing – review and editing. **Elliott Goff:** Methodology; software; supervision; writing – review and editing. **Eva Maria Wölfel:** Supervision; writing – review and editing. **Samuel Ghatan:** Data curation; formal analysis; writing – review and editing. **Charlotte Ejersted:** Resources; writing – review and editing. **Gisela Kuhn:** Project administration; writing – review and editing. **Ralph Mueller:** Funding acquisition; project administration; writing – review and editing. **Morten Frost:** Conceptualization; funding acquisition; project administration; writing – original draft; writing – review and editing.

## Conflict of Interest Statement

The authors do not have any conflicts of interest to declare.

### Peer Review

The peer review history for this article is available at https://www.webofscience.com/api/gateway/wos/peer‐review/10.1002/jbm4.10832.

## Supporting information


**FIGURE S1.** Sex stratified lacunar parameters for cort (A–F) and trab (G–L) regions. Data presented for male (m) and female (m) independently of MVD status (A–C, G–I) and separated for T2D and T2D complicated by MVD (D–F, J–L). A: Lacunar volume (Lc.V): *P* = 0.0.09, B: Lacunar sphericity (Lc.Sr): *p* = 0.51, C: Lacunar density (Lc.N/BV): *p* = 0.51, D: Lc.V: f: *p* = 0.201, M: *p* = 0.084, E: Lc.Sr: f: *p* = 0.686, M: *p* = 0.0016, F: Lc.N/BV: f: *p* = 0.803, m: 0.169, G: Lc.V: *p* = 0.38, H: Lc.Sr: *p* = 0.68, I: Lc.N/BV: *p* = 0.08, J: Lc.V: f: *p* = 0.201, m: *p* = 0.972, K: Lc.Sr: f: *p* = 0.686, m: *p* = 0.728, L: Lc.N/BV: f: *p* = 0.803, m: *p* = 0.803. N for cortical analysis: nine women (4 T2D + MVD), 24 men (9 T2D + MVD), for trabecular analysis: 10 women (5 T2D + MVD), 25 men (10 T2D + MVD, 9 T2D + MVD for LcN/BV).

**TABLE S1.** Sex stratified iliac crest cortical and trabecular morphometric osteocyte lacunar parameters. Data presented in median and IQR. *p‐*values were determined using Wilcoxon tests and linear regression models controlled for age.
