# Supplementary material for: Microvascular Disease Associates with Larger Osteocyte Lacunae in Cortical Bone in Type 2 Diabetes Mellitus
Source: JBMR Plus. 2023 Oct 27;7(11):e10832. doi: 10.1002/jbm4.10832 (PMC10652180; doi:10.1002/jbm4.10832)
Supplement: Supplementary file 2 — TABLE S1. Sex stratified iliac crest cortical and trabecular morphometric osteocyte lacunar parameters. Data presented in median and IQR. p‐values were determined using Wilcoxon tests and linear regression models controlled for age. [file JBM4-7-e10832-s001.docx]

Supplementary table 1: Sex stratified iliac crest cortical and trabecular morphometric osteocyte lacunar parameters. Data presented in median and IQR. *P-*values were determined using Wilcoxon tests and linear regression models controlled for age.

| **TRAB** | **FEMALE (n=10)** | **MALE (n= 25)** | **Beta** | **SE** | ***P*** |
| --- | --- | --- | --- | --- | --- |
| Lc.N [#] | 295,393 | 474,941 | - | - | - |
| Lc.V [µm^3^] | 185 (IQR 163-198) | 177 (IQR 154-193) | - | - | 0.38 (log) |
| Lc.Sr [-] | 0.749 (IQR 0.712-0.766) | 0.749 (IQR 0.736-0.763) | - | - | 0.68 (log) |
| Lc.N/BV [#/mm^3^] | 14,797 (IQR 12,779-17,332) | 12,659 (IQR 10,488-15,033) | - | - | 0.08 (log) |
| **CORT** | **FEMALE (n= 9)** | **MALE (n= 24)** | **Beta** | **SE** | ***P*** |
| Lc.N [#] | 2,399,342 | 4,729,426 | - | - | - |
| Lc.V [µm^3^] | 151 (IQR 146-155) | 165 (IQR 149-176) | - | - | 0.09 (log) |
| Lc.Sr [-] | 0.696 (IQR 0.694-0.698) | 0.701 (IQR 0.696-0.705) | - | - | 0.51 (log) |
| Lc.N/BV [#/mm^3^] | 30,126 (IQR 26,550-32,564) | 29,065 (IQR 26,318-32,623) | - | - | 0.51 (log) |
|  |  |  |  |  |  |
